# Job satisfaction and stressors for working in out-of-hours care – a pilot study with general practitioners in a rural area of Germany

**DOI:** 10.1186/s12875-018-0777-7

**Published:** 2018-06-22

**Authors:** R. Leutgeb, J. Frankenhauser-Mannuß, M. Scheuer, J. Szecsenyi, Katja Goetz

**Affiliations:** 1 Department of General Practice and Health Services Research, University Hospital Heidelberg, Marsilius-Arcades, Western Tower, Im Neuenheimer Feld 130.3, 69120 Heidelberg, Germany; 2 Headquarter of Control Centre, District Bergstraße, Gräffstrasse 5, 64646 Heppenheim, Germany; 3 Institute of Family Medicine, University Hospital Schleswig-Holstein, Campus Luebeck, Ratzeburger Alle 160, 23538 Luebeck, Germany

**Keywords:** Effort-reward imbalance, General practitioner, Health services research, Out-of-hours care, Job satisfaction

## Abstract

**Background:**

Challenging work environment, high workload, and increasing physician shortages characterize current rural general practice in Germany and in most European Countries. These factors extend into Out-Of-Hours Care (OOHC). However, little research about potential stressors for general practitioners (GPs) in OOHC settings is available. This pilot study aimed to evaluate workload, different elements of job satisfaction and stressors for GPs in OOHC and to analyze whether these aspects are associated with overall job satisfaction.

**Methods:**

Cross-sectional survey with a sample of 320 GPs who are working in OOHC was used to measure workload in OOHC, job satisfaction (using the Warr-Cook-Wall scale) and stressors with the effort-reward imbalance questionnaire. In order to assess associations between workload, job satisfaction and stressors at work we performed descriptive analyses as well as multivariable regression analyses.

**Results:**

The response rate was 40.9%. Over 80% agreed that OOHC was perceived as a stressor and 79% agreed that less OOHC improved job satisfaction. Only 42% of our sample were satisfied with their overall job satisfaction. The regression analysis showed that the modification of current OOHC organization was significantly associated with overall job satisfaction.

**Conclusions:**

Our results suggest that OOHC in the current form is a relevant stressor in daily work of rural GPs in Germany and one of the reasons for a decreasing overall job satisfaction. Strategic changes such as the implementation of structural reforms e.g. reducing frequency of OOHC duties for each GP and improving continuing professional development options related to OOHC are needed to address current workload challenges experienced by GPs providing OOHC in Germany.

## Background

Significant demographic changes in age distribution in the German population along with the desire of Generation Y physicians (millennium generation, born between 1980 and 2000) for a balanced work-life situation and the high workloads of general practitioners (GP) are all factors influencing the shortage of GPs, especially in rural areas. Health policy makers in Europe increasingly recognize that ‘primary care, the backbone of a nation’s health care, is at grave risk of collapse` a statement of the American College of Physicians in 2006 [1]. In the Netherlands, the United Kingdom and in Scandinavian countries health reform at the end of the 1990s and the early 2000s created more attractive working conditions for doctors working in primary care and providing Out-of-Hours Care (OOHC). In Germany, OOHC reforms are only just being begun at a political level [2–5].

In Germany, under the National Health Insurance Scheme, there are physicians who are assigned a catchment area to provide care for insured patients (in German: *Vertragsarzt*). In 2015, approximately 110,000 of these physicians who worked in regular care -thereof 35,100 GPs and 11,500 internists worked additional in OOHC, except for doctors’ with chronic diseases and in-patient physicians [6]. A recent study with German GPs views on the situation in OOHC reported on critical issues, in particular highlighting that OOHC is one primary factor making the role as a GP in Germany unattractive [7]. Unfortunately, there are no data available which show the workload of physicians who worked in OOHC and regular care.

Little published research has reported on working conditions and occupational demands in the workplace of OOHC physicians. Mc Loughlin et al. conducted a study in 2005 with GPs who were working in newly founded OOHC-Co-operatives in comparison to GPs not working in such Co-operatives. No differences were found regarding mental health and job stress between these two groups [8]. Two other studies revealed an improvement of quality of life for GPs working in Co-Operatives [8, 9].

Workload, job satisfaction and working conditions of physicians are crucial aspects for provided the quality of care [10]. This is an aspect not only in regular care but also in OOHC. Because of the shortage of GPs in many European countries (and in oversea countries like Australia) and the overcrowding of emergency departments in hospitals it is essential to improve the job satisfaction and working conditions of physicians [2, 11–14]. However, to date, only a few studies have explored workload, job satisfaction and stressors at work of GPs in primary care OOHC settings in Europe. Therefore, the aim of the study was to evaluate the workload, different elements of job satisfaction and stressors at work of GPs in OOHC with established survey instruments and to analyze whether these aspects are associated with overall job satisfaction concerning GPs working in OOHC rotation groups.

## Methods

### Setting

OOHC is defined as care during out-of-hours periods where regular medical ambulatory services are not available. In Hesse, a federal state of Germany where this study was performed, these periods extend from 7:00 p.m. to 7:00 a.m. on Monday, Tuesday and Thursday, and from 2:00 p.m. to 7:00 a.m. on Wednesday and Friday and also on weekends and public holidays [15]. The OOHC periods, especially on Wednesdays and Fridays, are scheduled as an agreement between the licensed physicians and the Association of Statutory Health Insurance Hesse. Since introduction of the national emergency number 116117 in 2015 the OOHC periods were adapted in all federal states of Germany. The characteristics of OOHC in rural areas of Germany in the year 2012 are cited in Table 1 [16]. The Organization for Economic Co-operation and Development (OECD) methodology classifies local administrative units (LAU) with a population density below 150 inhabitants per km^2^ as rural. It is defined intermediate, if the share of population living in rural LAU is between 15 and 50% and predominantly rural, if the share of population living in rural LAU is higher than 50%. Our survey was sent to all panel doctors in the specific local district ‘Landkreis Bergstraße’ of the federal state of Hesse. This LAU is ‘intermediate rural’ in terms of the OECD-definition [17].

**Table 1:** Out-Of-Hours Care (OHHC)-services in rural areas of different federal states of Germany in 2012 [16]

Service obligation for all panel physicians to do on-call duty but not to maintain registration as a GP or (as another specialist discipline) • Approximately 110,000 Most frequent model of OOHC in Germany in rural areas • OOHC rotation groups 30–50 physicians OOHC care centre • OOHC practices predominantly in the middle of the local district respectively care provision in GP practices • Either GPs of the region or hired clinicians work in the OOHC care centres Opening hours in OOHC centre • On weekdays From 07:00 pm to 07:00 am • On Wednesday already at 2:00 pm • From Friday 07:00 pm to Monday 7:00 am • On holidays Catchment area • Local districts with 40,000–80,000 inhabitants • Distance of patients to OOHC-centre 15-20 km Accessibility • Access via regional telephone numbers • about 10–15% walk in without a call in advance Telephone triage • In 2012 no triage model was implemented • The doctor himself answered the phone calls, rarer a nurse Provision of care • Telephone advice • Consultation-hours • Home visits.	

### Study design and participants

This exploratory study was based on a written questionnaire survey in one rural region in Germany. Data were collected from GPs who worked in OOHC. Between August 2012 and November 2012 all 320 GPs of the region were contacted to participate on the postal questionnaire survey. Addresses were selected via an address register of the Association of Statutory Health Insurance of Hesse, Germany. The GPs were invited to participate by mail. The return of the anonymous paper-based questionnaire was classified as informed consent. No reminder was sent out.

### Measurements

#### Workload in OOHC

The workload of GPs in OOHC was measured with a self-developed questionnaire based on qualitative interviews with GPs in OOHC and literature review. The interviews focused on the experiences of GPs who worked in OOHC. All GPs were interviewed with the same semi structured interview guideline. Theme saturation reached after 8 interviews. Within the 8 interviews the statements were categorized to individual stress, general conditions of care and present situation of care. The categories were transformed to items in an interactive process by an interdisciplinary team consisted of GPs who worked in OOHC, a sociologist and a health service researcher This questionnaire consists of 7 items rated on a four-point Likert scale (from 1 = fully disagree to 4 = fully agree). Cronbach’s α was 0.772 for the items of workload. The questionnaire was not validated before.

### Job satisfaction

For evaluation of job satisfaction a modified Warr-Cook-Wall job satisfaction scale was used, which has been already used in previous studies with GPs [18, 19]. The instrument consists of 9 items to different aspects on job satisfaction and 1 item to overall job satisfaction. These 10 items rated on a seven-point Likert scale (from 1 = extreme dissatisfaction to 7 = extreme satisfaction). A higher overall mean score indicates higher job satisfaction. An example item is: ‘How satisfied are you with your income? ’ Cronbach’s α was 0.855 for the job satisfaction scale without the general question of job satisfaction.

### Stressors at work

Stressors at work were measured with effort-reward imbalance (ERI) developed by Siegrist [20]. It is a well-known instrument which was validated in different human service settings and has been already used in previous studies with physicians [21, 22].This measurement consists of three scales: effort (6 items), reward (11 items) and overcommitment (6 items). Effort and reward as extrinsic components constitute the ERI. It means an imbalance between professional overspending and reward. The scale effort evaluates the professional overspending (e.g. working under high time pressure) and reward scale measures the reward at workplace like recognition for work or adequate remuneration. All questions could be rated on a five-point Likert scale ranging from 1 = low stress to 5 = high stress. The reward scale is subdivided in three subscales: ‘esteem‘ (5 items), ‘job security‘ (2 items), and ‘job promotion‘ (4 items). The effort-reward ratio (ER-ratio) was calculated based on the following equation: ER-ratio = 11 × effort/6 × reward. Values of ER-ratio over 1.0, a high amount of effort not met with adequate reward is indicated. The scale overcommitment as intrinsic component is independently from the ER-ratio and was assessed with 6-item on a four-point rating scale, from agree to disagree with the given statement. The Cronbach’s α was 0.830 for the effort-reward imbalance scale.

### Data analysis

Analyses were performed using SPSS version 22.0 (SPSS Inc., IBM). A descriptive analysis was performed concerning the 7 items of workload and the 10 items of the job satisfaction scale. Means, standard deviation and 95% confidence intervals as well as a summarization of the percentage of fully agree and agree respectively extreme satisfied, rather satisfied and satisfied of workload and job satisfaction were reported. The descriptive analysis of the scales of effort, reward and its subscales and overcommitment included means and standard deviation. Moreover, sum scores of the ERI as well the ER-ratio was calculated. The sum score of effort varies between 6 (no stress) and 30 (very stressful). The sum score of reward ranges between 11 (lowest reward) and 55 (high level of reward) [20]. For further analyses the full range of answer options and not the summarization from the variables were used. The dependent variable ‘overall job satisfaction’ as well as the independent variables were handled as linear variables. Pearson’s correlation was used to find out which the independent variables individual characteristics, workload, aspect of job satisfaction and effort, reward and overcommitment showed a significant correlation with the dependent variable ‘overall job satisfaction’. Afterwards, a linear regression analyses were used to explore potential associations between the dependent variable ‘overall job satisfaction with OOHC’ and independent variables which correlated significantly with the dependent variable. An alpha level of *P* < 0.05 was used for tests of statistical significance.

## Results

Three hundred and twenty questionnaires were handed out to GPs who worked in OOHC, 131 participated on the survey. The response rate was 40.9%.

Table 2 shows the characteristics of the participating OOHC physicians.

**Table 2:** Summary of the basis characteristics of the physicians involved in Out-Of-Hours Care (OOHC)

Characteristics		Our sample (*n* = 131)
Gender, n (%)	Male	99 (75.6)
	Female	32 (24.4)
Age, years; mean (SD), range (min – max)		51.8 (8.1), 32–70
OOHC-duties in the quarter; mean (SD)		4.4 (4.6)
Home visits during OOHC; mean (SD)		4.0 (4.0)
Number of patient during OOHC; mean (SD)		7.3 (7.9)
Telephone calls during OOHC; mean (SD)		7.4 (8.0)
Attending retirement homes during OOHC; mean (SD)		2.1 (3.1)
Attending nursing homes during OOHC; mean (SD)		1.9 (2.1)
Kilometer distance during OOHC; mean (SD)		19.6 (21.3)
Participating OOHC physicians within the district; mean (SD)		26.1 (17.2)
Quarterly contact group^a^, n (%)	< 500 patients	9 (6.6)
	500–1000 patients	19 (14.5)
	1001–1500 patients	55 (42.0)
	> 1500 patients	47 (35.9)

^a^n = varies due to missing data; *SD* standard deviation, *OOHC* Out-Of-Hours Care

Moreover, 26% of our sample stated that they felt ‘extreme stress due to care for people in retirement or nursing homes within OOHC’.

### Evaluation of workload and job satisfaction

The evaluation of workload in OOHC and job satisfaction scale is presented in Table 3. 79.4% of GPs agreed that job satisfaction could improve due to less OOHC, 80.9% agreed that working in OOHC was a general stressor. Furthermore, 72.5% of GPs were satisfied with ‘colleagues and fellow workers’ and 69.5% were satisfied with ‘freedom of working method’ but 27.5% were satisfied with ‘hours of work’ and 30.6% were satisfied with ‘income’.

**Table 3:** Descriptive statistics of workload and job satisfaction of the physicians involved in in Out-Of-Hours Care (n = 131)

Items of workload in OOHC^a^	Mean (SD)	CI 95%	Percentage of answers to fully agree and agree
Negative effects on job satisfaction due to OOHC	2.83 (1.0)	2.7–3.0	68.7
Psychosocial stress due to OOHC	3.06 (0.9)	2.9–3.2	73.3
Negative effects on the following day after OOHC	3.11 (0.9)	3.0–3.3	77.1
Improvement of general job satisfaction due to less OOHC	3.28 (0.9)	3.1–3.4	79.4
OOHC as a general stressor	3.19 (0.9)	3.0–3.3	80.9
Financial incentive to work more in the OOHC centre of the rotation groups	2.21 (1.0)	2.0–2.4	32.8
Modification of current OOHC-organization	3.14 (1.0)	3.0–3.3	72.5
Items of job satisfaction^b^	Mean (SD)	CI (95%)	Percentage of answers to extreme, rather and satisfied
Amount of variety in job	4.88 (1.5)	4.6–5.1	62.6
Opportunity to use abilities	4.89 (1.5)	4.6–5.2	64.2
Freedom of working method	5.05 (1.5)	4.8–5.3	69.5
Amount of responsibility	4.85 (1.5)	4.6–5.1	63.4
Physical working condition	4.58 (1.3)	4.4–4.8	48.9
Hours of work	3.60 (1.6)	3.3–3.9	27.5
Income	3.69 (1.6)	3.4–4.0	30.6
Recognition for work	4.76 (1.3)	4.5–5.0	60.4
Colleagues and fellow workers	5.28 (1.2)	5.1–5.5	72.5
Overall job satisfaction	3.98 (1.6)	3.7–4.3	42.0

^a^ranged from 1 “fully disagree” to 4 “fully agree”

^b^ranged from 1 “extreme dissatisfaction” to 7 “extreme satisfaction”

*OOHC* Out-Of-Hours Care, *SD* standard deviation, *CI* Confidence interval

### The effort-reward imbalance

The different scores of the ERI and their scales are presented in Table 4. The scale ‘effort’ was high (mean = 21.0) in contrast to a low level of ‘reward’ (mean = 23.6). Also low levels of the subscales of ‘reward’ were observed. The mean score of the ER-ratio was 1.7. Over 127 (94.7%) of the GPs in OOHC showed an ER-ratio over 1.0.

**Table 4:** Effort-reward imbalance of physicians involved in Out-Of-Hours Care (*n* = 131)

Scales (range; minimum to maximum)	Mean (SD)
Effort (6–30)	21.0 (5.2)
Reward (11–55)	23.6 (7.1)
Overcommittment (6–24)	14.7 (3.0)
Subscales of reward-scale (range; minimum to maximum)	Mean (SD)
Job promotion (4–20)	8.9 (3.5)
Esteem (5–25)	10.0 (3.0)
Security (2–10)	4.7 (2.0)
ER-Ratio^a^	1.7

^a^value > 1.0: imbalance between high effort and low reward

*SD* standard deviation, *ER-Ratio* Effort-reward ratio

### Factors associated with overall job satisfaction

The correlation showed that the variables of workload with the exception of the variable “financial incentive to work more often in the OOHC centre of the rotation groups” correlated strong with the dependent variable “overall job satisfaction”. For the different aspects of job satisfaction, a strong correlation to the dependent variable with exception of variable “colleagues and fellow workers” was also found. The scales ‘effort’, ‘reward’ and ‘overcommitment’ correlated strongly with the dependent variable “overall job satisfaction”.

No correlation was found for the different individual characteristics which were presented in Table 2.

The linear regression analysis of the independent variables workload and job satisfaction on the dependent variable overall job satisfaction of working in OOHC is shown in Table 5. The linear regression model explained more than 46% (R^2^~ 0.462) of the variance of the dependent variable ‘overall job satisfaction’. A higher agreement to modification of current OOHC-organization was associated with more job satisfaction. More variety in the job was associated with more job satisfaction.

**Table 5:** Associations of workload, different aspects of job satisfaction and scales of effort-reward imbalance of of physicians involved in Out-Of-Hours Care to outcome variable ‘overall job satisfaction’ (results of the linear regression analysis, under specification of standardized beta coefficient, α = 5%)

	Variables	β (*p*-value)
Items of workload in OOHC	Negative effects on job satisfaction due to OOHC	−0.111 (0.274)
	Psychosocial stress due to OOHC	−0.066 (0.601)
	Negative effects on the following day after OOHC	−0.020 (0.876)
	Improvement of general job satisfaction due to less OOHC	−0.043 (0.755)
	OOHC as a general stressor	−0.117 (0.410)
	Modification of current OOHC-organization	−0.278 (0.008)
Items of job satisfaction	Amount of variety in job	0.226 (0.048)
	Opportunity to use abilities	0.001 (0.994)
	Freedom of working method	0.068 (0.515)
	Amount of responsibility	0.162 (0.145)
	Physical working condition	0.006 (0.950)
	Hours of work	0.017 (0.861)
	Income	0.067 (0.503)
	Recognition for work	−0.033 (0.754)
Scales of effort-reward imbalance	Effort	−0.019 (0.860)
	Reward	0.077 (0.499)
	Overcommitment	−0.045 (0.647)
	Extreme stress due to care for people in retirement or nursing homes while OOHC	0.014 (0.877)
R^2^	0.462	

## Discussion

The aim of the current study was to evaluate workload, different elements of job satisfaction and stressors at work of GPs in OOHC and to explore potential associations to overall satisfaction. A comparison between our sample of participating GPs and the whole sample of GPs in Germany show similar results concerning age but differs slightly by gender, 24.4% women in our sample comparing to 43.9% in the whole sample of GPs in Germany [23]. It can be assumed that more men than women working as GP in OOHC which is comparable to studies concerning after-hours care in Australia [14, 24]. Our results showed that our participants were mostly satisfied with their colleagues but dissatisfied with their income and working hours. Over 80% of our sample agreed that working in OOHC was perceived as a general stressor. Moreover, GPs highly agreed with the statement: ‘less OOHC-duties could improve general job satisfaction’, which was also observed within the regression model and was strongly associated with overall job satisfaction. It could be assumed that the modification of current OOHC-organization could have an impact on a positive feeling at working in OOHC.

Our findings concerning workload and job satisfaction of GPs are in agreement with previous studies not only in different European countries but also in the USA and Australia [14, 19, 24–26]. In contrast to our study with low income satisfaction rate a study in Australia show a high level of income satisfaction in after-hours care; it can be explained as physicians were paid per patient [14].

A survey conducted by the Commonwealth Fund evaluated that German GPs have the highest workload with the most working hours per day, the shortest consultation time with their patients and were most unsatisfied with own professional situation in comparison to GPs in other Western European countries and the USA [26]. Additional, it was found that German physicians felt more in control of their working hours than British physicians but the impact on job satisfaction is unclear [27].

It could be assumed that high workload, dissatisfaction with income and obligations for duty in OOHC could be a reason for reducing the overall satisfaction of Germans GPs.

The health policy consequences of this assumption are potentially severe. The shortage of GPs, particularly in rural areas could be exacerbated. This is already a problem in many European countries [3, 4, 9, 28]. Considering our results about workload and job satisfaction, it could be assumed that our sample of physicians is increasingly less motivated to do the OOHC-duties. In the Netherlands, 85% of the GPs delegate 25% of their shifts, so most of the GPs do their shifts solely in GP- cooperatives. Like German physicians, they also complain about the high workload because of the large number of patients with minor ailments. However, they feel responsible to deliver continuity of primary care. Unlike the situation in Germany, GPs in the Netherlands have to provide OOHC to maintain their registration as a GP. This could be an additional explanation to the high quota of GPs in the Netherlands doing their shifts in OOHC [29].

The development in Germany is different, a high percentage of OOHC duties –exact figures are not available- are transferred to assistant doctors of hospitals and locum doctors. A key element, the continuity of care with experienced GPs in OOHC, is lost, which could have an impact on quality of care and should be examined in further studies. Campbell et al. argued that GPs have to lead OOHC services because of their generalized skills and experiences. Patients’ satisfaction with OOHC increases if they are treated by GPs [30]. In contrast to this statement, it can be assumed that patients would visit hospital emergency departments if they are dissatisfied with the treatment in primary OOHC because of the inexperience of the assistant doctors working there. The consequences would be further inefficiencies and overcrowding in the emergency departments and potentially rising costs for the health care system [31].

Kjaer et al. showed the importance of continuing professional development programmes for GPs to improve professional standards in general practice [32]. Therefore, in our opinion investment in continuing professional development related to OOHC could improve the quality of treatment in OOHC. For example, an interactive learning program including updates of new knowledge in clinical practice could be implemented for the medical staff (GPs, assistant physicians and nurses) and others practicing in OOHC. Additional training in the competencies related to triage, reasons of encounter in OOHC and the resulting therapy options would be desirable [33]. It can be assumed that continuing professional development, especially concerning collaborative skills between health professionals, in the implementation of validated triage systems and in the implementation of error managements in OOHC could increase the quality of care and could potentially positively affect workload and job satisfaction of physicians and other health care professionals working in OOHC. Experts of the European research network for out-of-hours primary health care (EurOOHnet) have discussed such strategies during their conferences in the recent years and highlighted their potential impact on job satisfaction in OOHC [34]. In particular, an international study (SAFE-EUR-OOH) started in 2014 under the leadership of the Norwegian colleagues’ to prove the safety attitudes questionnaire in OOHC in different member countries [35–37].

In our study population, a high imbalance between effort and reward could be observed, nearly 95% of the GPs in OOHC showed an ER-ratio over 1.0 (mean score 1.7). It was found that GPs from Sweden and Norway have a significant lower effort-reward-ratio as physicians in Germany explained by better working conditions in these countries [38, 39]. Finnish GPs feel more distressed than the Finnish specialists because of the perceived increasing demands in the subject of general practice [40]. Furthermore, in Japan it was observed that effort-reward imbalance of GPs was significant associated with depression [41]. Interestingly, for primary care physicians who work in after-hours care in Australia a low level of stress was observed [42]. Concerning the special situation of OOHC in the region of Germany examined, our results could indicate that the high ER-ratio of GPs working in OOHC is associated with low satisfaction regarding income, higher frequency of home and nursing home visits, and psychosocial stressors like the misusing of health care utilization in OOHC through non-urgent complaints. These aspects should be considered for potential health policy reforms in OOHC. It can be concluded that more research is needed to identify potential risk factors as reference points, which could be improved through reforms. It is evident that organizational and structural reforms should be developed to improve the balance between effort and reward and to reduce the health risks of GPs in OOHC. Unfortunately, studies about health risks of GPs in OOHC are rare. One study with GPs showed that higher job satisfaction is associated with good health behavior. It was also demonstrated that support from colleagues influences positively the work and health of GPs [43]. Therefore, it could be assumed that working in OOHC with support from colleagues as a source of social support prevents mental or physical illness [44]. Moreover, it has been observed that surveys of patient experiences with OOHC provide additional data about quality of care and working situation of GPs [45].

To our knowledge there are few research studies about workload, job satisfaction and potential stressors in a primary care OOHC setting that have been published to date. The present study used well-proven instruments, the Warr-Cook-Wall scale and the ERI, which enables comparison of results as they have been both validated and have often been used in other studies [18, 20]. However, the survey tools were not piloted and validated for this study. We only measured the internal consistency for each of the three survey tools: workload of GP’s in OOHC, job satisfaction and effort-reward-imbalance. Our sample may not be representative for all OOHC physicians in Germany because we only involved physicians in one rural area who were willing to participate voluntarily on the survey. But a response rate of 40.9% is notably high and one of the strength of this study in comparison to the statement by Kelley et al. [46]. They assumed for postal questionnaire surveys a response rate of 20% as normal for such surveys [46].

A limitation is that we could not evaluate all possible key factors like family situation, leisure opportunities or infrastructure in the local district ‘Landkreis Bergstraße’, which could contribute to GPs perceptions of overall job satisfaction. Furthermore, the demographic data presenting in Table 2 are subjective statements made by the participating physicians. Official data from the “Association of Statutory Health Insurance Physicians” are not available. Unfortunately, we did not define clearly within the demographic questions what kind of OOHC shift we meant. Furthermore another limitation is that our presented data of this pilot study were from 2012 and should be examined in a new research project with a longitudinal study design in consideration of the current health reform in OOHC in Germany and in comparison to rural and urban regions. Moreover, this is a cross-sectional study and thus, we must be cautious to derive causal links from these findings. Significant results might be due to chance and will need to be confirmed in further targeted studies. Moreover, there are no clear statements in the literature concerning the statistical analysis of surveys using Likert scales [47, 48]. Therefore, we handled the Likert scales as an interval which could implicated a potential statistical bias.

## Conclusions

The study concludes that our study sample perceived working in OOHC as a general stressor and show low satisfaction with income and working hours. Moreover, less duties in OOHC could increase the overall job satisfaction of GPs and could lead possibly to lower ER-ratio. Our results might support the modification of the current organization of OOHC in the region of Germany studied and could have implications for other regions in Germany. Moreover, it can be recommended that for future health care delivery in OOHC it is important to invest in a continuing professional development. Further research should explore whether the implementation of training programs with the focus on how to deal with the frequently minor ailments of patients in OOHC, how to deal with for example launched triage systems and how to deal with error management could improve the quality of care and could resulted in an improvement of job satisfaction of GPs in OOHC.

## Abbreviations

ERI: Effort-reward imbalance
EurOOHnet: European research network for out-of-hours primary health care
GP: General Practitioner
LAU: Local Administrated Units
OECD: Organization for Economic Co-operation and Development
OOHC: Out-Of-Hours Care
SD: Standard deviation
